# MicroRNA-29b Contributes to Collagens Imbalance in Human Osteoarthritic and Dedifferentiated Articular Chondrocytes

**DOI:** 10.1155/2017/9792512

**Published:** 2017-05-22

**Authors:** David Moulin, Véronique Salone, Meriem Koufany, Thomas Clément, Isabelle Behm-Ansmant, Christiane Branlant, Bruno Charpentier, Jean-Yves Jouzeau

**Affiliations:** ^1^Laboratoire d'Ingénierie Moléculaire et Physiopathologie Articulaire (IMoPA), UMR 7365 CNRS-Université de Lorraine, Biopôle de l'Université de Lorraine, Campus Biologie-Santé, 9 avenue de la Forêt Haye, BP 20199, 54505 Vandœuvre-lès-Nancy Cedex, France; ^2^Département de Pharmacologie Clinique et Toxicologie, Centre Hospitalier Universitaire, Hôpital Central, 29 avenue du Maréchal de Lattre-de-Tassigny, 54035 Nancy Cedex, France

## Abstract

**Objective:**

Decreased expression of collagen type II in favour of collagen type I or X is one hallmark of chondrocyte phenotype changes in osteoarthritic (OA) cartilage. MicroRNA- (miR-) 29b was previously shown to target collagens in several tissues. We studied whether it could contribute to collagen imbalance in chondrocytes with an impaired phenotype.

**Methods:**

After preliminary microarrays screening, miR-29b levels were measured by RT- quantitative PCR in* in vitro* models of chondrocyte phenotype changes (IL-1*β* challenge or serial subculturing) and in chondrocytes from OA and non-OA patients. Potential miR-29b targets identified* in silico* in 3′-UTRs of collagens mRNAs were tested with luciferase reporter assays. The impact of premiR-29b overexpression in ATDC5 cells was studied on collagen mRNA levels and synthesis (Sirius red staining) during chondrogenesis.

**Results:**

MiR-29b level increased significantly in IL-1*β*-stimulated and weakly in subcultured chondrocytes. A 5.8-fold increase was observed in chondrocytes from OA versus non-OA patients. Reporter assays showed that miR-29b targeted COL2A1 and COL1A2 3′-UTRs although with a variable recovery upon mutation. In ATDC5 cells overexpressing premiR-29b, collagen production was reduced while mRNA levels increased.

**Conclusions:**

By acting probably as a posttranscriptional regulator with a different efficacy on COL2A1 and COL1A2 expression, miR-29b can contribute to the collagens imbalance associated with an abnormal chondrocyte phenotype.

## 1. Introduction

MicroRNAs (miRNAs) are a family of small (20- to 22-nt) endogenous noncoding RNAs. Since their discovery in* Caenorhabditis elegans*, hundreds of potential miRNAs have been discovered in animals. However, we are just in the process of establishing their precise roles in biological mechanisms [1]. Expression of mammalian miRNAs includes several steps. They are most frequently produced from long precursors (pri-miRNAs) by the sequential action of two RNase III-like enzymes: in nuclei, Drosha produces ~70-nucleotide pre-miRNA hairpin precursors, which are exported to the cytosol by exportin 5, where they are converted into miRNA:miRNA^*∗*^ duplexes by the Dicer RNase. The duplexes are then taken in charge by the RNA-induced Silencing Complex (RISC) [2], allowing their association with their partner sequences in the 3′-untranslated region (3′-UTR) of targeted messenger RNAs (mRNAs). As a consequence, either translation of the targeted mRNA is repressed or it is degraded (reviewed in [3]). Such regulation by miRNAs is expected to concern one-third of mammalian mRNAs [4]. Furthermore, the expression patterns of miRNAs are cell, tissue, and development-stage specific [5], so that miRNAs play a key role in cell differentiation and development, as exemplified by studies on skeletal development [6]. Accordingly, microarray technology and deep-sequencing approaches have revealed correlation between dysregulation of miRNA expression and several pathological conditions [7] as well as disease progression. Nevertheless, further studies are needed to identify miRNA variations which are specifically associated with pathological conditions in order to demonstrate whether miRNAs can be used as disease biomarkers [8] and/or as modulators of pathologies [9].

Osteoarthritis is a highly prevalent joint disease [10] characterized by a progressive destruction of the extracellular matrix (ECM) of articular cartilage, and contributions by synovium, subchondral bone, and other joint tissues to the disease onset are now well-established [11]. During OA, chondrocytes, which are the only functional cell type of cartilage, become unable to maintain the dynamic equilibrium between synthesis of cartilage-specific components and their enzymatic degradation. The main consequences are altered cell-matrix interactions favouring occurrence of an abnormal chondrocyte phenotype and concomitant cartilage destruction [12]. As a consequence, OA cartilage is characterized by a decreased expression of cartilage-specific genes and occurrence of biomarkers accounting for loss of the differentiated phenotype or switch towards hypertrophy [13]. In this line, profiling studies identified chondrocytes miRNAs that were expressed differentially in human OA cartilage [14–16], some of which being shown to regulate matrix degradation such as miR-27b, miR-140, and miR-199a [17–20]. However, the overlap of miRNA patterns between studies was quite low [14–16], possibly as the result of different disease durations and severities, cartilage regions studied, or demographic features of healthy controls [21]. Indeed, it is worth underlining that several risk factors have been identified for OA, such as ageing, genetic predisposition, and environmental factors including obesity and diabetes or biomechanical constraints. As some miRs have been identified as being responsive to one or either of these factors, such as miR-222 or miR-365 for biomechanics [22, 23] and miR-103 or miR-143 for obesity [24], it is not surprising that miRs profiles may vary between OA patients depending on their OA phenotypes [25]. As a consequence, miRs profiling has not been performed to date on cohorts being large enough to be representative of all OA clinical phenotypes. We therefore performed a “bench to bedside” experiment by looking first to variations of miRNAs expression in controlled cellular models of human chondrocyte phenotype loss and then by controlling the relevance of one of them as a possible biomarker of OA-associated phenotype changes. Amongst the 43 miRs varying in either IL-1*β*-induced or subculture-induced chondrocyte dedifferentiation, we focused on miR-29b, which is known to target various collagens thereby contributing to fibrosis of soft tissues [26]. We showed that miR-29b level increased in a panel of OA patients matched for age, sex, and BMI with non-OA controls. We showed further its ability to target COL2A1 more efficiently than COL1A2 3′-UTR, while leaving COL10A1 3′-UTR unaffected in a gene-reporter system. Using the ATDC5 chondrogenic cell line we also demonstrated that the stable overexpression of pre-miR-29b reduced the synthesis of fibrillar collagens while provoking a major accumulation of COL2A1 and a weak accumulation of COL1A2, but neither of COL1A1 nor of COL10A1, mRNAs. These data suggest that miR-29b targets mainly COL2A1 in chondrocyte and induces translational suppression. As the other collagens studied are poorly affected, our data strongly support a contribution of miR-29b upregulation to the loss of the differentiated chondrocyte phenotype associated with OA.

## 2. Patients and Methods

### 2.1. Patients

Cartilage samples were obtained from patients undergoing knee or hip arthroplasty in conformity with the declaration of Helsinki principles and after procurement of a written informed consent (see Table S1 in Supplementary Material available online at https://doi.org/10.1155/2017/9792512). All OA patients (*n* = 18) were evaluated by an orthopaedic surgeon and fulfilled the clinical and radiological criteria of the American College of Rheumatology for knee [27] or hip [28] OA. OA lesions corresponded to the grade III or IV of the Kellgren/Laurence radiographic criteria for assessment of OA. Non-OA chondrocytes were obtained from 5 age-, sex-, BMI-, and anatomical site-matched patients with joint break or in the context of multiorgan procurements (Table S1).

### 2.2. Isolation and Culture of Human Chondrocytes

Chondrocytes were obtained from human cartilage samples as described elsewhere [29]. Briefly, cartilage samples were washed twice in sterile phosphate buffered saline (PBS) and then cut into small pieces. Chondrocytes were isolated after a sequential digestion of the extracellular matrix with pronase (0.15%, w/v) for 2 h and collagenase (0.2%, w/v) overnight at 37°C. After centrifugation, cells were suspended in Dulbecco's Modified Eagles Medium/Ham's F-12 (DMEM/Ham's F-12) supplemented with 10% (v/v) fetal calf serum (FCS), 2 mM L-glutamine, penicillin (0.1 U/ml), streptomycin (100 ng/ml), and 250 ng/ml amphotericin B (Invitrogen, Cergy-Pontoise, France) and then seeded as primary chondrocytes culture in 75 cm^2^ culture flasks at a high density (2 × 10^4^ cells/cm^2^). They were cultured as monolayers until confluence (10–12 days) in a humidified atmosphere containing 5% CO_2_, with change of culture medium every 3-4 days. Confluent primary chondrocytes were then either challenged with human recombinant interleukin-1*β* (R&D Systems) or serially subcultured (see below for details).

### 2.3. *In Vitro*-Induced Loss of Differentiated Articular Chondrocyte Phenotype

The loss of differentiated chondrocyte phenotype was induced by 2 complementary methods [30, 31]. On one hand, chondrocytes from non-OA donors were stimulated for 48 h with 10 ng/ml of hrIL-1*β* (R&D Systems). On the other hand, chondrocytes were subcultured as monolayers on plastic substrata for 3 passages to favour a switch towards a fibroblast-like phenotype. Both methods have relevance to reproduce changes in phenotype biomarker expression reported in OA chondrocytes [32] including aggrecan, COL2A1, COL2B, MMP-9, and MMP-13, which were evaluated by RT-qPCR.

### 2.4. miRNA (miR) Profiling

Total RNAs were isolated from cell monolayers with the miRCURY™ RNA Isolation kit (Exiqon), before treatment with the RQ1 RNase-Free DNase (Promega) according to the manufacturer's protocol. After tests for RNA quality and the presence of small RNAs with a 2100 chip bio-analyzer™ (Agilent technologies), the samples were labelled and hybridized with miRCURY LNA Arrays (v.11.0* hsa, mmu, and rno*) coated with probes for 1376 human miRs (Exiqon).

### 2.5. Real-Time Quantitative PCR (RT-qPCR)

To quantify mRNA expression of target genes, RT-PCR analysis was performed as previously described [33], using specific set of primers (Table S2). The mRNA level of each gene of interest and of the ribosomal protein RP(S)29, chosen as housekeeping gene in chondrocyte [34], was determined three times. Quantifications of gene expression were performed using standard curves of purified PCR products with concentrations ranging from 10^−3^ to 10^−9^ ng/ml. Results were expressed as the ratio between the mRNA level of the gene of interest and that of the RP(S)29 gene.

TaqMan™ MicroRNA assays (Applied Biosystems), performed according to manufacturer's protocols, were used for real-time quantitative PCR evaluation of the miR-29b level. Each experiment was run in triplicate. Quantification of relative miRNA expression levels was determined by the 2^−ΔΔCT^ method [35]. Using BestKeeper software [36], microRNAs with a low expression variance in chondrocytes and ATDC5 cells were identified and used as endogenous controls.

### 2.6. Constructs for Luciferase Reporter Assays and microRNA-29b Expression

The pGL3-Luc::hCOL2A1, pGL3-Luc::hCOL1A2, and pGL3-Luc::hCOL10A1 plasmids were built by cloning full length human COL2A1, COL1A2, or COL10A1 mRNA 3′-UTRs into the pGL3 Luciferase Reporter Vector (Promega), respectively. The 3′-UTRs were amplified from HeLa cell genomic DNA using the following primers: GACTCTAGAGGACCCAAGTACTTTCCA (Forward) and GGCCGTCTAGATGTACTTTCCAATAATCT (Reverse) for COL2A1, GGACTCTAGACTTGTGGCTTTTGAATATC (Forward), and GCCGTCTAGAAACAAATGCTGAATCTG (Reverse) for COL1A2, GGACTCTAGAGTACACACAGAGCTAATC (Forward) and GCCGTCTAGAAACAAATGCTGAATCTG (Reverse) for COL10A1. Amplified DNA fragments were purified using the NucleoSpin™ Extract II Kit (Macherey-Nagel), and digested overnight at 37°C with* Xba *I restriction endonuclease (Fermentas) in the Tango buffer 10X. Digested DNA fragments were gel purified and ligated into the* Xba *I-digested pGL3 vector to generate pGL3-Luc::hCOL2A1, pGL3-Luc::hCOL1A2, and pGL3-Luc::hCOL10A1 plasmids (reporter plasmids, Figure 3(a)). Control plasmids were produced containing the putative miR-29b seed sequences in a reverse orientation (antisens, AS). Mutant plasmids for hCOL1A2 or hCOL2A1 3′-UTRs were produced by PCR-directed mutagenesis of the putative miR-29b seed sequences (Figure 4(a)).

The pcDNA3.1-pre-miR-29b and pcDNA3.1-pre-miR-199a (chosen as a IL-1 responsive control) plasmids were used to express miR-29b and miR-199a from their human precursors, hsa-pre-miR-29B1 (81 bp sequence, referred to as MI0000105 in miRbase v17) and hsa-pre-miR-199a-1 (71 pb sequence, referred to as MI0000242 in miRbase v17), respectively. The hsa-pre-miR-29B1 and hsa-pre-miR199a-1 DNA sequences were amplified from HeLa cell genomic DNA using the GACGGTACCCTTCAGGAAGCTGGT (Forward) and GACGGATCCCCCCCAAGAACACTG (Reverse) and the GACGGATCCGCCTAACCAATGT (Forward) and GACGGTACCGCCAACCCAGTGTT (Reverse) primer pairs, respectively. Amplified DNAs were purified and digested for one hour at 37°C with* Kpn *I and* Bam*H I restriction endonucleases (Fermentas) in the Tango buffer 1x. Digested DNAs were gel purified and inserted in the pcdNA 3.1 vector (Invitrogen) cleaved by* Kpn *I and* Bam*H.

### 2.7. Transfection of HeLa Cells for Luciferase Reporter Assays

HeLa cells were transfected with 500 ng of pGL3-Luc::hCOL2A1, pGL3-Luc::hCOL1A2, or pGL3-Luc::hCOL10A1 plasmid and 500 ng of pcDNA3.1-pre-miR-29b or pcDNA3.1-pre-miR199a or empty pcDNA3.1 and 5 ng of the pRL plasmid encoding* Renilla* luciferase (Promega) using EXGEN500™ (Euromedex) in 12-well plates. Triplicate measures of the luciferase activity were performed 24 h after transfection, using the Dual-Luciferase Reporter Assay System™ (Promega) and Xenius XL™ luminometer (Safas) equipped with double injectors. The* firefly* luciferase activity was normalized to* Renilla *luciferase activity. Each experiment was repeated at least 5 times.

### 2.8. Culture of ATDC5 Cells in Monolayer and Micromass

ATDC5 cells were supplied by the ECACC Cell Bank (Sigma). Undifferentiated cells were maintained at 37°C in a humidified atmosphere containing 5% CO_2_ in T25 flasks filled with DMEM/F12 medium supplemented with 10 *μ*g/ml human transferrin (Sigma), 3 × 10^−8 ^M sodium selenite (Sigma), 100 units/ml penicillin, 100 *μ*g/ml streptomycin, 0.5 *μ*g/ml amphotericin B, and 5% fetal bovine serum (Invitrogen). After reaching 70% of confluence, monolayers were trypsinized and cells were replated in 12-well plates at a density of 2 × 10^4^ cells/well before chondrogenic differentiation by insulin supplementation according to [37]. In some experiments, ATDC5 cells were cultured in micromass as described by [38] with cell differentiation promoted as described above.

### 2.9. Generation of microRNA-29b Overexpressing ATDC5 Stable Transfectants

Undifferentiated ATDC5 cells were transfected with 500 ng of empty pcDNA3.1 or pcDNA3.1-pre-miR-29b using EXGEN500 (Euromedex). Two days later, cells were diluted 10-fold then incubated in complete DMEM/F12 medium supplemented with 400 mg/ml geneticin (GIBCO BRL). After 2 weeks, drug-resistant clones were picked up and expanded. Cells were then plated in 12-well plates or cultured in micromass in the chondrogenic conditions described above. Five experiments were performed on independent pools of clones (both in monolayer and micromass).

### 2.10. Sirius Red Staining of ATDC5 Cells

ATDC5 cells that had been cultured for 14 days in 6-well plates were washed twice with PBS and fixed with 95% ethanol for 30 min. The fixed cells were stained with 1% Sirius red (Sigma, [39]) for 5 min before two washings with H_2_O. Sirius red, a strong anionic dye, stains collagen by reacting, via its sulphonic acid groups, with basic groups present in the collagen molecule. The elongated dye molecules are attached to the collagen fibre in such a way that their long axes are parallel.

### 2.11. Statistical Analysis

Data were expressed as mean ± SEM. Comparisons were performed by one-way analysis of variance (ANOVA) followed by Tukey post hoc test for multiple samples or Student's* t-*test for comparison between two samples using GraphPad Prism 5. Differences were considered as significant if *P* value was less than 0.05.

## 3. Results

### 3.1. Microarray Screening of miRNAs in* In Vitro* Models of Chondrocytes Phenotype Loss

As shown in Table 1, the challenge of non-OA chondrocytes with IL-1*β* modified significantly the miRNA expression profile in the ]0.8; 1.2[-fold changes interval. Ten miRs were upregulated with the highest level seen for miR-146a (Table 1(a)). In contrast, 9 miRs were downregulated with the lowest expression level observed for miR-199a (Table 1(b)). In favour of the predictive relevance of our cellular model to OA-associated chondrocyte changes, the steady-state levels of several miRs previously reported to be affected in OA chondrocytes, that is, miR-146a, miR-140, miR-455, and miR-199a, displayed significant variations in our screening assays. Furthermore, seven miRs varied similarly upon serial culturing of chondrocytes as compared to IL-1*β* treatment (data not shown). As expected, mRNA quantifications of genes representative of the chondrocyte phenotype indicated the reduced expression of the extracellular matrix components (aggrecan and collagen types IIA and B) and an increased expression of the matrix metalloproteinases MMP-9 and MMP-13 in IL-1*β*-stimulated chondrocytes (Supplementary Figure S1).

### 3.2. Increased Expression of miR-29b in Human OA Chondrocytes

**Figure 1:**
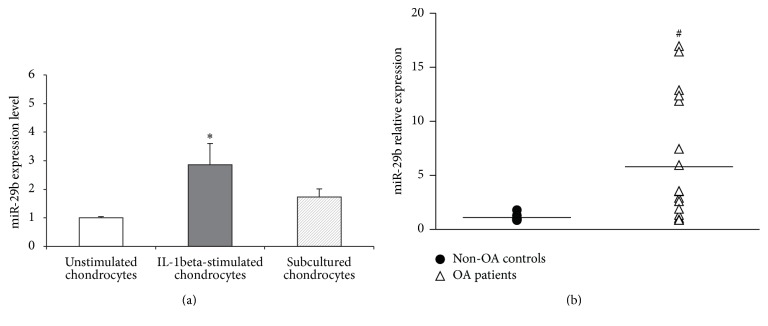
Variation of miR-29b level in human chondrocytes with an altered phenotype. (a) Chondrocytes stimulated with 10 ng/ml of hrIL-1*β* for 48 h (*n* = 5) or subcultured repeatedly on plastic dishes (*n* = 3). (b) Chondrocytes from OA patients undergoing joint arthroplasty (*n* = 18). Quantification was performed by the 2^−ΔΔCT^ method using the invariant expression of miR-103, miR-423-3p, and miR-191 as endogenous controls. Data are presented as relative levels with reference to non-OA controls (*n* = 5) arbitrarily considered as having a value of 1. Bars show the mean ± SEM. ^*∗*^*P* < 0.05 versus unstimulated chondrocytes; ^#^*P* < 0.05 versus chondrocytes from non-OA controls (*n* = 5).

A more precise estimation of the miR-29b level in our cell models was performed by quantitative RT-PCR. MiR-29b level was more markedly increased after IL-1*β* challenge (averaged 2.8-fold) than after serial culturing (averaged 1.7-fold) (Figure 1(a)). Very interestingly, a significant increase of miR-29b level (averaged 5.8-fold) was also observed in chondrocytes obtained from OA patients compared to non-OA donors (Figure 1(b)). Unexpectedly, RT-PCR analysis showed no significant differences between OA and non-OA patients for aggrecan, collagens IIA and IIB, and collagen X mRNA levels due to large scattering of values in the OA population (data not shown). Nonetheless, the averaged collagen type X mRNA level was higher in OA patients. Altogether our data are consistent with miR-29b upregulation being associated with an overall loss of the dedifferentiated chondrocyte phenotype.

### 3.3. Identification of Putative mRNA Targets of miR-29b in Chondrocytes

**Figure 2:**
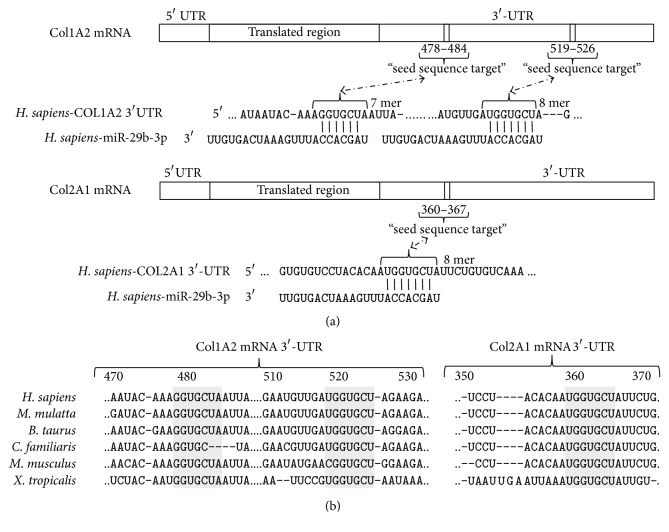
Putative sequences targeted by the miR-29b seed sequence in the 3′-untranslated region (3′-UTR) of human COL1A2 and COL2A1 mRNAs. (a) Predicted duplex formation between miR-29b and 3′-UTRs by the TargetScan algorithm. (b) Cross-species conservation of the targeted sequences in the 3′-UTRs of COL1A2 and COL2A1 mRNAs.

From the profiling study, target mRNAs were selected from miRNA-mRNA target pairs predicted by at least 2 of the 3 MicroCosm Targets, TargetScan, and PicTar algorithms. Amongst the 3′-UTRs of mRNAs known to be expressed in human OA chondrocytes [40–42], the 3 algorithms confirmed those encoding chains of the fibrillar collagens COL2A1 (collagen type II, *α*1[II]), COL1A1 (collagen type I, *α*1[I]), and COL1A2 (collagen type I, *α*2[I]) as possible targets of the miR-29b seed sequence. The probability scores were very high (free energy −21.6 kcal/mol for COL2A1) with at least one putative (Figure 2(a)) and phylogenetically conserved (Figure 2(b)) binding site in each UTR. MiR-29b was shown previously to target directly COL2A1 mRNA-3′-UTR in mouse mesenchymal stem cells [43] and COL1A1 and COL1A2 mRNA-3′-UTRs in hepatic stellate cells [44]. Noteworthily, no target sequence for miR-29b was identified in the 3′-UTR of COL10A1 mRNA. As the level of miR-29b was enhanced in our* in vitro* models of phenotype loss, we anticipated that miR-29b might contribute to the abnormal collagens profile reported in OA chondrocytes.

### 3.4. Direct Targeting of Human Chondrocyte Collagens mRNAs-3′-UTRs by miR-29b

**Figure 3:**
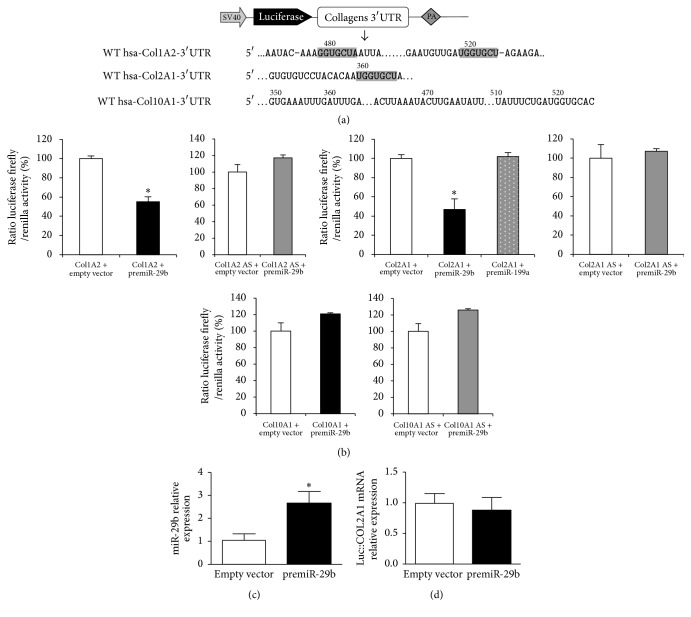
Study of miR-29b interaction with human collagens mRNAs using luciferase reporter assays. (a) Schematic construct of pGL3-collagens reporter. SV40: SV40 promoter, PA: polyadenylation signal, and WT: wild-type. The sequences predicted to be targeted by the seed sequence of miR-29b in the 3′-UTRs of human COL1A2 or COL2A1 mRNAs are highlighted. 3′-UTR of human COL10A1 is used as a control lacking any predicted target sequence and represented arbitrarily in the same sequence location as those targeted in hCOL1A2 or hCOL2A1. (b) Luciferase activities in HeLa cells cotransfected either with pcDNA3.1-pre-miR-29b or a negative control (pcDNA3.1-empty), and the internal control plasmid encoding* Renilla* luciferase (RLuc), and either pGL3-Luc::hCOL1A2 nor pGL3-Luc::hCOL2A1 or pGL3-Luc::hCOL10A1 sens or antisens (AS) constructs. Cotransfection with either pcDNA3.1-pre-miR-199a or a negative control (pcDNA3.1-empty) was used as a nonspecific control of IL-1*β* effect. Data are shown as relative luciferase activity, taking the activity measured in HeLa cells transfected with plasmid pcDNA3.1-empty as the reference 100% value for each construct. (c) miR-29b level determined by the 2^−ΔΔCT^ method as described in Figure 2. (d) Luciferase::hCOL2A1 mRNA level determined by quantitative PCR and normalized to RPS29 mRNA chosen as a housekeeping gene. Data are expressed as relative levels, taking the mRNA level in HeLa cells transfected with plasmid pcDNA3.1-empty as the reference value 1. In all experiments, bars show the mean values ± SEM of at least 3 independent experiments, each being run in triplicate. ^*∗*^*P* < 0.05 versus control.

**Figure 4:**
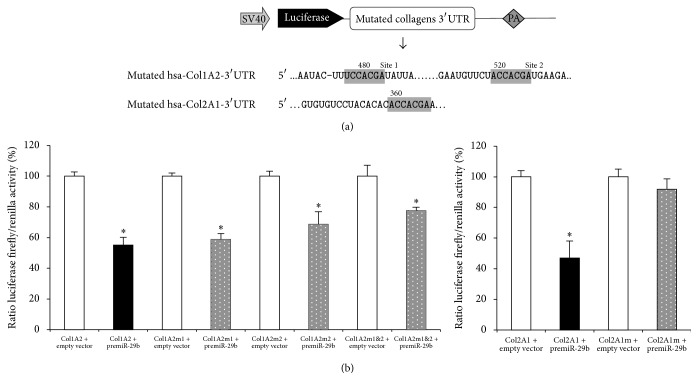
Consequence of mutation of the miR-29b targeted sequences in human COL1A2 or COL2A1 mRNAs on luciferase reporter assays. (a) Schematic construct of pGL3-collagens reporter. SV40: SV40 promoter, PA: polyadenylation signal. In the 3′-UTRs of human COL1A2 or COL2A1 mRNAs, the 8 bps mutation made in the sequences predicted to be targeted by the seed sequence of miR-29b are highlighted. (b) Luciferase activities in HeLa cells cotransfected either with pcDNA3.1-pre-miR-29b or a negative control (pcDNA3.1-empty), and the internal control plasmid encoding* Renilla* luciferase (RLuc), and either pGL3-Luc::hCOL1A2, pGL3-Luc::hCOL1A2 mutated in site 1 (m1), site 2 (m2), or both (m1&2), or pGL3-Luc::hCOL2A1 or pGL3-Luc::hCOL2A1-mutant. Data are shown as relative luciferase activity, taking the activity measured in HeLa cells transfected with plasmid pcDNA3.1-empty as the reference 100% value. In all experiments, bars show the mean values ± SEM of at least 3 independent experiments, each being run in triplicate. ^*∗*^*P* < 0.05 versus control.

Then, we checked for the direct interaction of human miR-29b with the 3′-UTRs of human COL2A1 and COL1A2 in HeLa cells, using a 3′-UTR luciferase-based reporter assay and COL10A1 as a “negative” control (Figure 3(a)). This cell type was chosen because of its low level of expression of both miR-29b and collagens mRNAs (data not shown). Moreover, COL1A2 was chosen over COL1A1 because it was more affected by miR-29b overexpression in hepatic stellate cells [44]. In cells cotransfected with premiR-29b, the luciferase activity was decreased by 2.1-fold for hCOL2A1 3′-UTR and 1.8-fold for hCOL1A2 3′-UTR whereas no effect was observed for hCOL10A1 3′-UTR (Figure 3(b)). Control experiments showed a significant expression of mature miR-29b in our system (Figure 3(c)) and no significant decrease of the mRNA level of the chimeric transcripts Luc::hCOL2A1 upon miR-29b overexpression (Figure 3(d)). When the cotransfection assay was repeated with pGL3-Luc plasmids mutated in their putative target sequences for miR-29b (Figure 4(a)), no significant loss of luciferase activity was detected for hCOL2A1 3′-UTR (Figure 4(b)). In contrast, a significant 1.3- to 1.7-fold decrease of luciferase activity was observed for hCOL1A2 3′-UTR with the less marked reduction being noted for the dual mutation of miR-29b putative target sequences (Figure 4(b)). In this experimental system, no reduction of hCOL2A1 3′-UTR luciferase activity was noted when cells were transfected with premiR-199a (Figure 4(b)) chosen as an IL-1*β*-inducible control [17]. Taken together, these data strongly suggest that miR-29b interacted directly with the 3′-UTR of human COL2A1 mRNA and less markedly with the 3′-UTR of COL1A2.

### 3.5. Translational Inhibition of Collagens Expression in a Chondrogenic Cell Line Stably Overexpressing miR-29b

**Figure 5:**
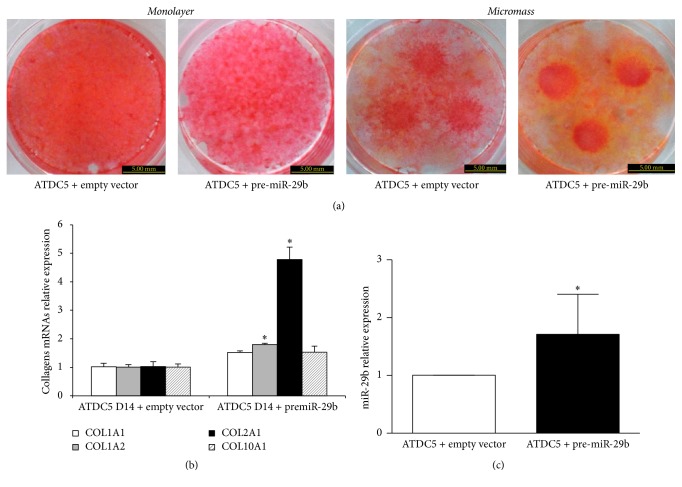
Effect of a stable miR-29b overexpression on ATDC5 cells phenotype during chondrogenesis. Undifferentiated cells were transfected with pcDNA3.1-pre-miR-29b or a negative control (pcDNA3.1-empty) and then cultured for 14 days in chondrogenic conditions. (a) Representative pictures of Sirius red staining of monolayer (left) or micromass (right) cultures. (b) COL1A1, COL1A2, COL2A1, and COL10A1 mRNA levels normalized on RPS29 mRNA determined by RT-quantitative PCR. (c) Expression of miR-29b mRNA relative to miR-21 (invariant endogenous control) determined by the 2^−ΔΔCT^ method. Data are presented as relative levels where control cells were allocated arbitrarily the value 1. Bars show the mean value ± SEM of 3 independent experiments, each of them being run in triplicate. ^*∗*^*P* < 0.05 versus control.

Recombinant plasmids encoding either the human pre-miR-29b (pcDNA3.1-pre-miR-29b) or the pcDNA3.1-empty vector as a negative control were used to generate stably transformed chondrogenic ATDC5 cell lines. These transformed cells were grown during 14 days in monolayers or in micromass. As shown in Figure 5(a), ATDC5 cells overexpressing miR-29b exhibited a decreased Sirius red staining compared to controls, whatever the culture system used during chondrogenic differentiation. At this time point, mRNA levels increased by 4.8-fold for COL2A1 and 1.8-fold for COL1A2, whereas COL1A1 or COL10A1 mRNAs levels remained below 1.5-fold variation (Figure 5(b)). No significant changes of collagens mRNAs were noted on day 21 between transformed and control cells (data not shown). A control experiment confirmed that miR-29b expression was doubled in the stably transfected cells (Figure 5(c)). These data show that in our experimental conditions a doubling of miR-29b level was sufficient to misregulate collagen synthesis in ATDC5 cells and suggests that collagen type II dysregulation was related to the translational step rather than to the transcriptional one, or to mRNA stability.

## 4. Discussion

Depending on cartilage layer and disease severity, OA chondrocytes have been reported to display enhanced apoptosis, insufficient anabolic activity, increased production of matrix degrading enzymes, and alteration of the chondrocyte phenotype [32]. A differentiated articular chondrocyte phenotype is classically defined by the ability to synthesize cartilage-specific molecules, especially aggrecan and some collagen subtypes [45]. Amongst them, collagen type II is highly expressed in activated functional chondrocytes and is a hallmark of phenotype maintenance [46]. Nonetheless, the expression of these well-established extracellular matrix biomarkers varies with cartilage depth [47] and biomechanical constraints [48] and during age-related focal cartilage lesions [49]. As a consequence, any scientist characterizing primary chondrocyte population obtained from OA patients has to face varied phenotypes with heterogeneity originating both from cartilage samples and patient history. Such heterogeneity may hamper identification of disease-related gene expression patterns and comparative analysis of miRNAs profiles in large-scale studies on primary OA chondrocytes [14–16, 40]. Therefore, we took advantage of well-established* in vitro* models of loss of the differentiated chondrocyte phenotype [31], namely, inflammatory cytokine challenge and repeated subculturing, to search for miRNA changes that might have relevance to OA-associated phenotype changes [32]. These culture models do not reproduce the extracellular matrix environment surrounding chondrocytes in cartilage and 3D culture systems are well known to preserve or even to restore the differentiated chondrocyte phenotype [50], as exemplified by expression or reexpression of collagen type II. This choice may be a possible drawback of our experimental strategy that we tried to limit by using first-passage chondrocytes from non-OA patients. We also considered that culture conditions forced the cells to lose their differentiated phenotype towards a more pathological one and that our primary objective was to look for differences between both states. Consequently, we think that the* in vitro* models we selected have relevance as screening tests for chondrocyte phenotype changes that can be observed in OA chondrocytes and offer the opportunity to limit heterogeneity of the biological response. Using this approach, we report here a strong increase of miR-146a and a significant decrease of miR-140, miR-199a, and miR-455 which are in good agreement with previous studies in OA chondrocytes [17–20, 51, 52]. These results validated the fact that our screening approach was not unfounded for identifying miRs changes associated with an OA-like cell phenotype.

Based on the collagens switch widely reported either in dedifferentiated [50] or OA [32] chondrocytes, we next focused our studies on miR-29b. Indeed, miR-29b level increased in both models of chondrocyte phenotype loss and it is thought to target several collagens in soft [26] or hard [43, 53] tissues. Members of the miRNA-29 family are highly conserved between human, mouse, and rat and the identity of their seed regions suggests large overlapping of their mRNA target sites [54]. They have been reported to target several extracellular matrix-related genes [54], in particular collagens [53, 55, 56], and their silencing [55] or their downregulation by TGF-*β* [56, 57] is thought to promote fibrosis or systemic sclerosis [56]. We failed to detect any significant variations of miR-29a or c in our preliminary miRNA profiling study. This observation is in good agreement with data from previous miRNA profiling studies performed on human OA chondrocytes, since a limited downregulation of miR-29a was reported in only one of the three experiments [14–16]. Moreover, by microarray analysis, only miR-29b was reported recently to increase very early (day 1) in the knee joints of mouse induced for OA by destabilization of the medial meniscus (DMM) [53]. Using the RT-qPCR technique, the expression of miR-29a, miR-29b, and miR-29c was reported to be increased in both chondrocytes from OA patients and femoral cartilage of mouse in the hip avulsion injury model [53]. However, a differential expression of miR-29 members has been reported [55] as a consequence of transcription of their precursors from two distinct genomic* loci* and variable posttranscriptional degradation [58]. In addition, although mature miRs were reduced by TGF-*β*1 in human chondrocytes, their precursors were affected variably by culture conditions suggesting a differential regulation at the level of their maturation [53]. We therefore focused our effort on miR-29b although we cannot exclude that changes in miR-29a and miR-29c expression could have been observed using the more sensitive RT-qPCR technique for their screening.

*In silico* analysis suggested 3′-UTRs of human COL1A2 and COL2A1, but not of COL10A1, as putative targets of miR-29b-3p with the highest probability score obtained for the miR-29b-hCOL2A1 pair. Noteworthy, mutation of the target sequence in the COL2A1 3′-UTR completely reversed the decreased luciferase activity provoked by pre-miR-29b whereas a partial reversal was observed for mutations of the two COL1A2 3′-UTR target sequences. This result demonstrates that COL2A1 is a main direct target gene of miR-29b. Although we did not perform gain- or loss-of-function experiments in chondrocytes, the increased level of miR-29b in both models of chondrocyte phenotype loss has to be considered with the known changes of collagens expression in these models. On one hand, IL-1*β* is well known to decrease collagen type II expression in primary or immortalized articular chondrocytes [59]. On the other hand, the phenotype switch induced by serial monolayer culture on uncoated surface is associated with a decreased level of COL2A1 and an increased level of COL1A1 mRNAs [50, 60]. Therefore, our data are consistent with miR-29b being a negative regulator of collagen type II expression in chondrocytes. Sox-9, a master regulator of chondrocyte differentiation [61], was shown to downregulate miR-29b both during chondrogenesis from a murine mesenchymal stem cell line [43] and when it was transiently overexpressed in human SW1353 chondrosarcoma cell line [53]. Sox-9 expression is downregulated by interleukin-1 [62] but was previously reported not to correlate with chondrocyte dedifferentiation during* in vitro* cultivation [63]. Thus, further studies are required to investigate whether the control of COL2A1 by miR-29b depends on Sox-9 expression changes in chondrocytes.

Our data led us to suggesting that miR-29b could, at least in part, contribute to the control of the collagen switch in chondrocyte. We found a significant increase of miR-29b expression in human OA chondrocytes that was consistent with its ability to target COL2A1 very efficiently and COL1A2 partially, while leaving COL10A1 unaffected in the luciferase assays. However, there was a large scattering of miR-29b levels and we failed to find any correlation with collagen type II or type I or X mRNA levels in OA chondrocytes. One has to mention that inverse correlations between miR levels and their target genes in cartilage have rather been reported in gain- or loss-of-function experiments with mimics or inhibitors compared to directly on biological samples [17–20, 43, 53]. Although these experiments are conclusive for the functional role of miRs, they cannot recapitulate the entire pathological context. In addition, the regulation of genes targeted by miRs, especially those encoding extracellular matrix components, involves numerous biological signals in OA such as cytokines or growth factors that can affect both miR levels and genes expression independently of miRs. This can, for example, be the case for IL-1*β* and TGF-*β*1 that are thought to play a pathological role in OA and are also able to modulate directly COL2A1 at the gene level through MAP kinases [64] or Sp1 [65] signaling pathways. Nonetheless, changes of miR-29b level in joint tissues may have some pathological relevance to the OA joint since miR-29b was also reported to promote osteoblast differentiation [66] and to favour mineral deposition in cells achieving terminal differentiation [67] and possibly cellular senescence [68].

As a consequence of the general inhibitory effect of miRNAs on mRNA translation, about 80% of the targeted mRNAs are subjected to an accelerated degradation process which explains the observed decreased mRNA level [69]. However, about 20% of the mRNA can be “translationally” inhibited by miRNAs without significant degradation [69]. Although miR-29b was shown to downregulate mRNAs for COL2A1 in mouse chondrocytes [43], for COL1A1 in MC3T3 osteoblasts [66], and for COL1A1 and COL3A1 in human aortic smooth muscle and adventitial fibroblasts [70], we report increased levels of COL2A1 and COL1A2 mRNAs in ATDC5 cells overexpressing miR-29b. This increase contrasted with the reduced procollagens synthesis shown by Sirius red staining when transformed cells were cultured either in monolayer or in micromass. Although this staining does not allow the distinction between collagen subtypes it becomes informative when faced with mRNAs changes. Indeed, fibrillar collagens are characterized by a right-handed triple helix structure made of [*α*_1_(II)_3_] chains for collagen type II and [*α*_1_(I)_2_*α*_2_(I)] chains for collagen type I. We found a major increase of COL2A1 mRNA coding for *α*_1_(II) chain whereas only a small increase was observed for COL1A2 mRNA coding for *α*_2_(I) chain and no accumulation was seen for COL1A1 mRNA coding for *α*_1_(I) chain. It is therefore highly probable that the reduced staining for total collagens accounted for the defective production of the *α*_1_(II) chains. Yan et al. [43] reported that COL2A1 mRNA level decreased in mouse chondrocytes transiently transfected with a miR-29a/b mimic, while it increased in ATDC5 cells transiently transfected with miR-29a/b inhibitors [43]. Such discrepancy may be, at least in part, due to differences in the experimental conditions used. Firstly, we performed a stable transfection to induce endogenous pre-miR-29b production and not a transient transfection with a miR-29-a/b mimic. A major consequence is that the miR29-a/b mimic was probably mainly located in the cytoplasm, whereas the overproduced pre-miR-29b recapitulates the physiological processing of miR-29b from the nucleus. Therefore, the subcellular localization and availability of mature miR-29b into the chondrocyte might greatly differ between both techniques with a subsequent impact on the probability of miR-29b to pair with target sequences in the 3′UTR of collagens mRNAs. Secondly, we allowed ATDC5 cells to differentiate for 14 days, in accordance with the chondrogenic differentiation process reported by Shukunami et al. [37], whereas ATDC5 cells were placed for only 2 days into chondrogenic medium in the study of Yan et al. [43]. This short-term exposure may be sufficient to stimulate expression of phenotype markers but is timely more consistent with activation of growth factors signaling pathways. Therefore the amount of COL2A1 to be targeted by miR-29b was likely much higher in our stably transfected ATDC5 cells than in the transiently transfected cells of Yan et al. The major increase of COL2A1 mRNA and the weak increase of COL1A2 but not of COL10A1 mRNAs were in line with the efficacy of premiR-29b in our luciferase assay. As day 14 corresponds to the exponential phase of chondrogenesis and collagen type II synthesis in the ATDC5 cell line [37], the accumulation of COL2A1 mRNA strongly suggests its reduced translation efficiency by miR-29b. Such possible translational suppression is consistent with the weak inhibitory effect reported for miR-29b on COL1A1 mRNA level in the IMR-90 lung fibroblast cell line, which contrasted the 3- to 5-fold variations observed at the protein level [71].

In conclusion, this work demonstrates that miR-29b is overexpressed in dedifferentiated and OA chondrocytes and targets selectively COL2A1 over COL1A2 but not COL10A1. By acting probably as a posttranscriptional inhibitor with a different efficacy on COL2A1 and COL1A2 expression, it can contribute to the collagens imbalance associated with an abnormal chondrocyte phenotype.

## Supplementary Material

Main demographic features of the OA or non-OA patients whose cartilage samples were used as primary sources of articular chondrocytes.

## Figures and Tables

**(a):** 

miRNA	IL-1*β* stimulated chondrocytes (fold-change)	Unstimulated chondrocytes (fold-change)	Ratio IL-1*β* stimulated/unstimulated
miR-146a	1.64	0.52	3.16
miR-155	1.36	0.57	2.38
miR-147b	1.22	0.67	1.83
miR-183	1.59	0.91	1.73
miR-146b-5p	1,32	0.77	1.71
miR-let7i	1.14	0.79	1.44
miR-29b	1.39	1.00	1.39
miR-886-5p	0.73	0.53	1.37
miR-568	1.14	0.87	1.32
miR-495	1.00	0.81	1.24

**(b):** 

miRNA	IL-1*β* stimulated chondrocytes (fold-change)	Unstimulated chondrocytes (fold-change)	Ratio of IL-1*β* stimulated/unstimulated
miR-23a	0.69	1.19	0.58
miR-140-5p	1.07	1.42	0.75
miR-455-3p	1.12	1.48	0.75
miR-140-3p	1.11	1.47	0.76
miR-199a-5p	0.94	1.24	0.76
miR-455-5p	1.05	1.36	0.77
miR-602	0.97	1.24	0.79
miR-1184	0.98	1.25	0.79
miR-199a-3p	1.14	1.42	0.80
