# Supplementary material for: MicroRNA-29b Contributes to Collagens Imbalance in Human Osteoarthritic and Dedifferentiated Articular Chondrocytes
Source: Biomed Res Int. 2017 May 22;2017:9792512. doi: 10.1155/2017/9792512 (PMC5458373; doi:10.1155/2017/9792512)
Supplement: Supplementary file 1 — Main demographic features of the OA or non-OA patients whose cartilage samples were used as primary sources of articular chondrocytes. [file 9792512.f1.pdf]

Figure S1

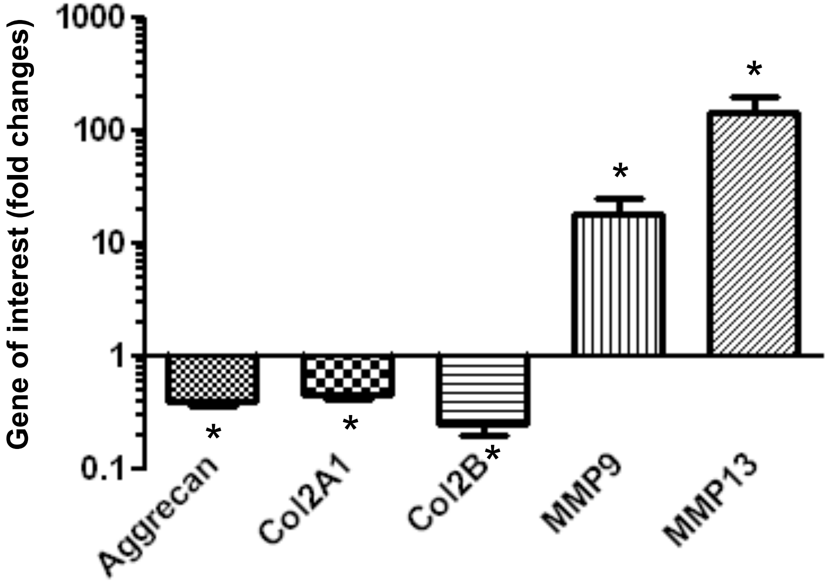

1000
100
10
1
0.1
Gene of interest (fold changes)
*
*
*
*
*
Aggrecan
Col2A1
Col2B
MMP9
MMP13

Table S1

Patients	Category	Age (years)	Gender	BMI (kg/m^2)	Cartilage source
1	Control	38	M	31	Knee
2	Control	39	M	27	Hip
3	Control	78	F	28	Knee
4	Control	83	F	18	Hip
5	Control	75	M	34	Hip
Mean/group	-	62.6	2 F/3 M	27.4	2 Knee/3 Hip
6	OA	62	F	29	Knee
7	OA	68	F	23	Knee
8	OA	63	F	25	Knee
9	OA	64	F	28	Hip
10	OA	69	F	29	Hip
11	OA	60	F	27	Hip
12	OA	62	F	23	Hip
13	OA	56	F	28	Hip
14	OA	79	F	24	Hip
15	OA	63	F	28	Hip
16	OA	60	M	26	Knee
17	OA	55	M	26	Knee
18	OA	69	M	29	Hip
19	OA	69	M	24	Hip
20	OA	57	M	21	Hip
21	OA	63	M	25	Hip
22	OA	54	M	30	Hip
23	OA	54	M	29	Hip
Mean/group	-	62.5	10 F/8 M	26.5	5 Knee/13 Hip

Table S2

Gene	Primer Sequence	Size (bp)	Tm (°C)
human ACAN	Sens: 5'-TCGAGGACAGCGAGGCC-3'<br>Antisens: 5'-TCGAGGGTGTAGCGTGTAGAGA	84	63
mouse COL1A1	Sens: 5’-GTCCCGCTGGTCAAGATG-3’<br>Antisens: 5'-CTCCAGCCTTTCCAGGTTCT-3’	120	59
human COL2A1	Sens: 5'-GGCCAGGATGTCCAGGAGGC-3'<br>Antisens: 5'-GGGCAGATGGGGCAGCACTC-3'	197	60
mouse COL2A1	Sens: 5’-TGGTATTCCTGGAGCCAAAG-3’<br>Antisens: 5'-ACCAGTTGCACCTTGAGGAC-3’	103	59
human COL2B	Sens: 5'-TCTGCCACGAGGTCCAGGGG-3'<br>Antisens: 5'-AGGGCCAGGATGTCCGGCAA-3'	176	65
mouse COL10A1	Sens: 5’-TTCATCCCATACGCCATAAAG-3’<br>Antisens: 5'-AGGGACCTGGGTGTCCTC-3’	103	57
human MMP9	Sens: 5'-AGGGACCACAACTCGTCATC-3'<br>Antisens: 5'-CATCGTCATCCAGTTTGGTG-3'	141	58
human MMP13	Sens: 5'-TGGTGGTGATGAAGATGATTTG-3'<br>Antisens: 5'-TCTAAGCCGAAGAAAGACTGC-3'	161	57
human RPS29	Sens: 5'-CAGACACGACAAGAGCGAGA-3'<br>Antisens: 5'-GGGTCACCAGCAGCTGTACT-3'	69	63
Mouse mS29	Sens: 5'-GGAGTCACCCACGGAAGTT-3'<br>Antisens: 5'-GCCTATGTCCTTCGCGTACT-3'	131	59
